# Development of effective 3D digital models for first‐time learners of musculoskeletal anatomy

**DOI:** 10.1002/ase.70220

**Published:** 2026-03-25

**Authors:** Alexander H. Safir, Michele M. Bird, Mary E. Orczykowski

**Affiliations:** ^1^ School of Kinesiology University of Michigan Ann Arbor Michigan USA; ^2^ Division of Anatomical Sciences University of Michigan Medical School Ann Arbor Michigan USA

**Keywords:** 3D model, anatomical education, course materials, digital resources, musculoskeletal anatomy, online learning, open source

## Abstract

Musculoskeletal anatomy is a critical component of allied health curricula. With the ubiquity of technology in the classroom and the recent COVID‐19 pandemic creating accessibility barriers for students, there is a need for viable digital resources to enhance learning by supplementing traditional textbook studying. This article describes the creation of an annotated, interactive, three‐dimensional digital model and presents preliminary data on its effectiveness for students learning musculoskeletal structures of the hip and knee for the first time. The 3D model was developed in Blender using open‐source files and was uploaded to the Sketchfab platform. Eighty‐one students in the musculoskeletal anatomy course at a large midwestern university took an assessment to measure their baseline anatomical knowledge, studied the testable structures from either the model or textbook images for 10 min, and took a follow‐up assessment. Students in the 3D Model Group saw greater increases from their baseline scores and also reported higher confidence in what they had learned, increased ability to visualize anatomical structures, and greater enjoyment of their resource than students who used textbook images. The findings presented here suggest that creating effective, accessible 3D digital resources is feasible for educators without training in technology‐related fields and that having access to these resources can be beneficial to first‐time learners of anatomy.

## INTRODUCTION

The widespread use of digital learning tools predates the COVID‐19 pandemic. Online learning management systems such as Canvas and Blackboard (now under the parent company “Anthology”) began the process of digitizing higher education as early as 1997.^1^, ^2^ Nevertheless, the idea of only having access to online educational resources was made salient by the quarantine policies implemented around the globe during the COVID‐19 pandemic in 2020. For those whose education necessitates resources that typically can only be accessed in person, especially across the pre‐health and allied health professions, there is a need for additional methods of reinforcing material learned via traditional lectures and textbook readings.^3^

The study of anatomy requires both strong spatial reasoning and the ability to visualize structures in three dimensions. A randomized controlled trial found that students who demonstrated strong mental imagery, visuospatial ability, and capacity for mental rotation performed better on a functional anatomy exam, concluding that focusing on the development of these abilities could improve a novice anatomy student's learning.^4^ One commonly used method to develop these skills is anatomical donor dissection, the historical gold standard for effective education in the field.^5^ However, various ethical and financial circumstances (in addition to world events, like the COVID‐19 pandemic) may prevent a student from accessing this valuable resource.^6^ Additionally, those interested in studying anatomy during their undergraduate education may not have access to the dissection labs found in graduate‐level courses. Within the framework for developing effective educational curricula, the loss of this critical laboratory experience confines students to lower levels of learning in Bloom's taxonomy.^7^ Rather than analyzing the relationship between the body's interconnected systems in a tangible setting, they are limited to memorizing facts off a standardized, two‐dimensional page that does not incite the development of mental imagery and rotation skills or visuospatial ability. Thus, a viable and highly accessible learning tool that allows the student to interact with anatomical structures in three dimensions would be a valuable supplement to traditional textbooks and lecture‐style learning, especially in the absence of anatomical dissection.

During the global quarantine caused by the COVID‐19 pandemic, educators developed a plethora of novel resources to enhance remote learning and to develop students' anatomical knowledge with limited or no use of in‐person dissection labs. One comprehensive review written during the height of the pandemic highlights some of these resources, which include 3D printed models, extended reality technologies (such as augmented reality), and a variety of other digital tools.^8^ While many of these resources are both technically impressive and effective learning supplements, they often have accessibility barriers at some stage of the creation and distribution process. The development of digital tools typically requires significant financial undertaking, including the cost of high‐end industrial 3D printers, photogrammetry scanners, high‐quality cameras, and powerful computers, which could limit reproducibility.^9^, ^10^ Some cost‐effective, individually licensed resources have been developed for anatomical education, such as Elsevier's Complete Anatomy ($75/year), but the burden of the cost falls directly on the student, further adding to the significant price of higher education.^11^ Additionally, the necessity of installing an app poses accessibility barriers in computer compatibility and processing power and requires some degree of technological literacy. However, despite the barriers presented by the creation and use of these resources, the review found that many technological tools can be equally effective to traditional anatomy curricula.^8^ These results demonstrate a need for digital learning resources which can be developed at a low cost and distributed to students with variable types of devices and experience with technology.

The development process and outcomes of providing students with digital, 3D anatomy models, specifically, have not been explored to a significant extent since the large‐scale shift to technology‐based learning caused by the initial COVID‐19 lockdown in 2020. Some of the studies and literature reviews which evaluate the use of 3D resources (whether they produce favorable or unfavorable results) may be outdated, as the prevalence– and quality– of technology in educational institutions has significantly increased over the past decade.^12^, ^13^, ^14^, ^15^, ^16^, ^17^ Additionally, many of these studies have been conducted within allied health graduate programs (medical, dental, veterinarian), with students whose prior exposure to anatomical education may be variable and potentially confounding.^9^, ^12^, ^14^ It would therefore be beneficial to study the implementation of 3D digital learning resources in undergraduate curricula, as many of these students are first‐time learners of anatomy.

Finally, previous studies in the *Anatomical Sciences Education* journal, a prolific publisher of novel educational techniques, have separately investigated either technology‐based methods of learning musculoskeletal anatomy or 3D digital models' role in comprehensive anatomy courses.^18^, ^19^, ^20^ However, a database search revealed that few studies have developed and evaluated 3D digital models for learning musculoskeletal structures (search keywords included “3D digital models,” “digital learning,” “digital resources,” “musculoskeletal,” “musculoskeletal anatomy,” “online,” and “online resources”). In this article, we explore the development of a free, browser‐based musculoskeletal model for undergraduate students and gather preliminary data on its potential as a resource for first‐time learners of musculoskeletal anatomy. We predicted that students studying musculoskeletal anatomy from the annotated, interactive, 3D digital anatomical model would see a greater increase in score between a pre‐ and post‐test compared to those using traditional, illustrated textbook images. Additionally, we predicted that students using the 3D model would report greater enjoyment of their resource, consistent with other literature which has measured this qualitative aspect.^12^, ^13^

## DESCRIPTION

### Context of model creation

In order to create a learning tool with the fewest possible accessibility barriers to both the developers and students, it was necessary that the creation and distribution of the model was performed at no or low cost, and with a procedure that avoided difficult technological tasks, such as performing original mesh work in a 3D modeling software. We searched for open‐source, downloadable files of musculoskeletal structures to be the building blocks for our 3D model. Open‐source files were chosen because they are free to view, download, and use for any student or educator who can benefit from them, saving the model's developers both money and the time of manually creating each musculoskeletal structure in a modeling software. The 3D creation suite, Blender, was chosen as the tool for assembling the model because it too is free to use and has a vast wealth of instructional videos and articles available online. The model was uploaded to Sketchfab, as the platform is free (with paid options for creators), browser‐based, and easy to learn. Additionally, Sketchfab is accessible on almost any type of modern device, including computers, tablets, and smartphones; however, it should be noted that navigating 3D space is most immediately intuitive when performed with a mouse and keyboard.

### Model creation

**FIGURE 1:**
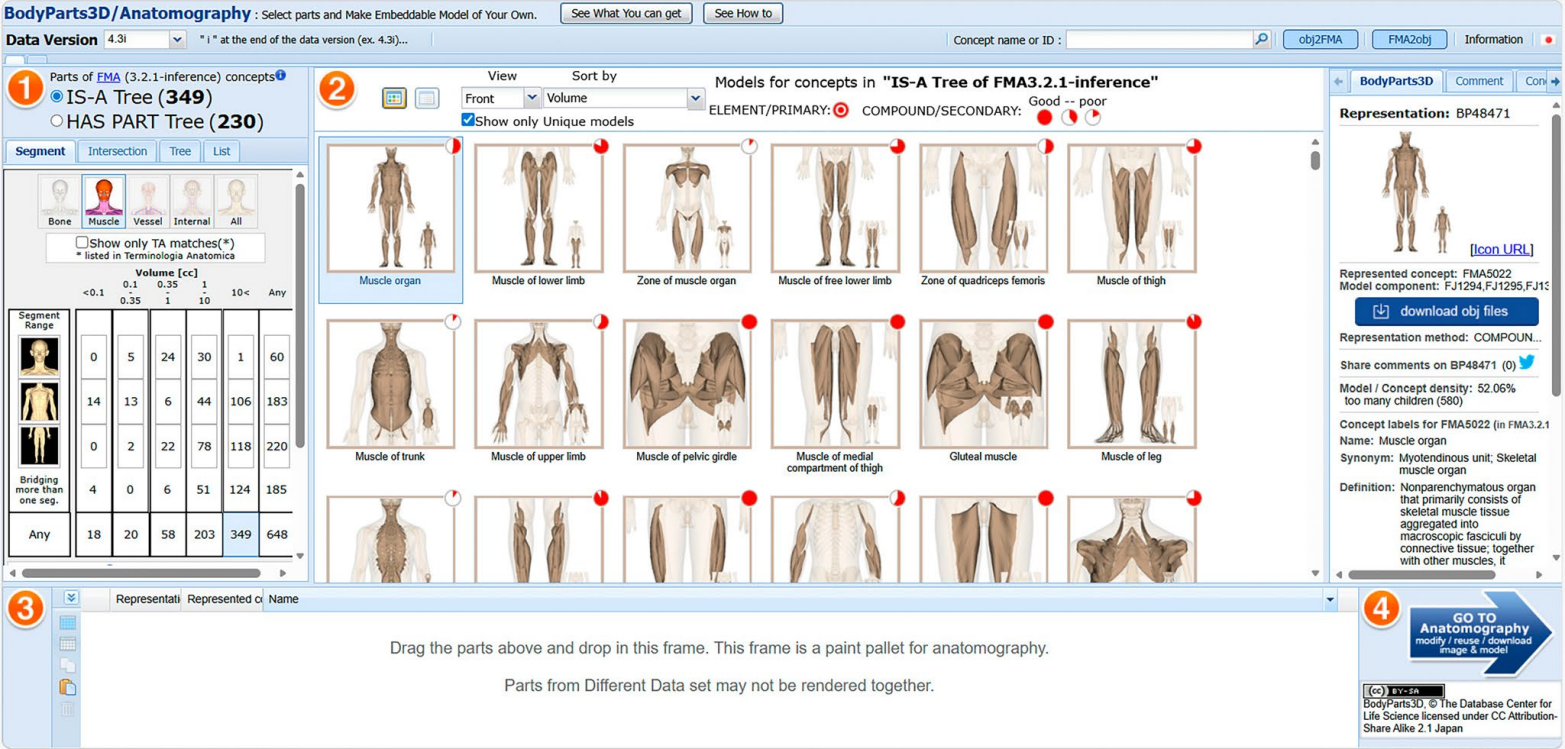
The BodyParts3D/Anatomography website, filtered to show the muscular structures in the 4.3i dataset.

**FIGURE 2:**
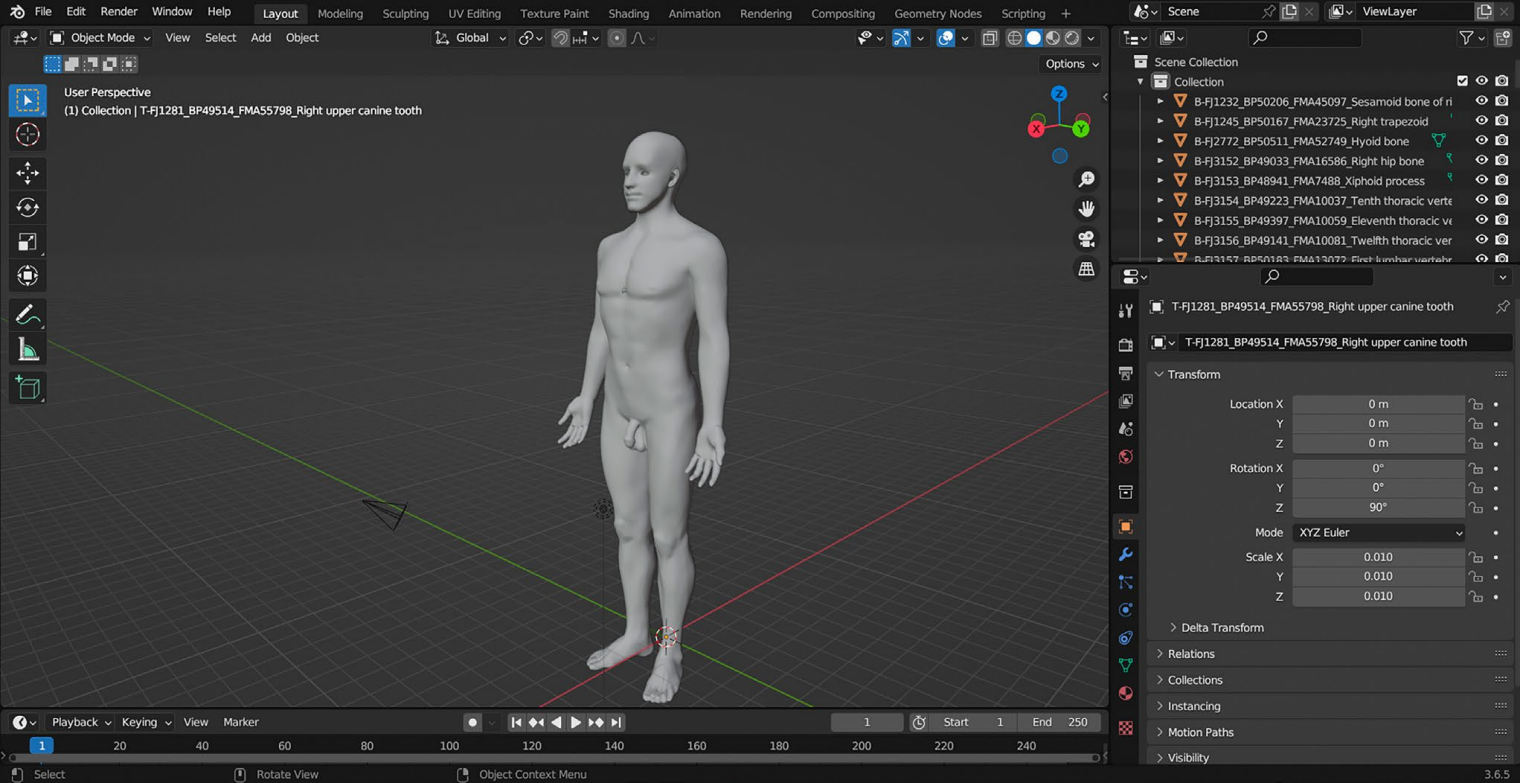
Initial import of files into Blender, viewed using the “solid mode” viewport shading.

Creation of the 3D anatomical model was performed in Blender, using free, MRI‐derived, open‐source files of musculoskeletal structures downloaded from the BodyParts3D/Anatomography website's 4.3i dataset (Figure 1).^21^ After downloading, the .obj files were all imported into Blender simultaneously, at a scale of 0.010, to reduce the file size and processing load (Figure 2). This generated a consolidated model complete with all the necessary musculoskeletal structures for an undergraduate course. Within Blender, the files were sorted into categories for organizational purposes, including “skin,” “bone,” “muscle,” and “cartilage.” Material properties were added to each category to distinguish them from each other and provide a viewing experience comparable to that of other educational resources (bones were made opaque white, cartilage a pale blue, etc.). Additionally, light sources (called “suns” in Blender) were added to the scene to improve visibility.

**FIGURE 3:**
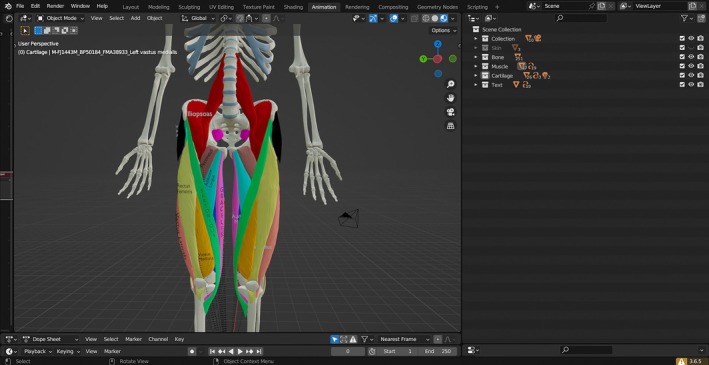
Objects have been sorted into easily navigable folders and materials have been assigned to each muscle—viewed in the “rendering” viewport shading.

To reduce visual clutter and accurately compare the 3D model to how the undergraduate musculoskeletal anatomy course is taught, it was determined that one day's (lecture/homework) worth of content should be included in the final model. The muscles of the hip and knee were selected for their relative complexity, layering, and novelty to many students, compared to more “recognizable” muscles found in the upper limb, like the biceps brachii. These muscles were each assigned their own material (color) and labeled using Blender's text option (Figure 3). All other muscle files were deleted from the model, leaving behind only the twenty‐one muscles that students in our pilot trial would be responsible for learning.

**FIGURE 4:**
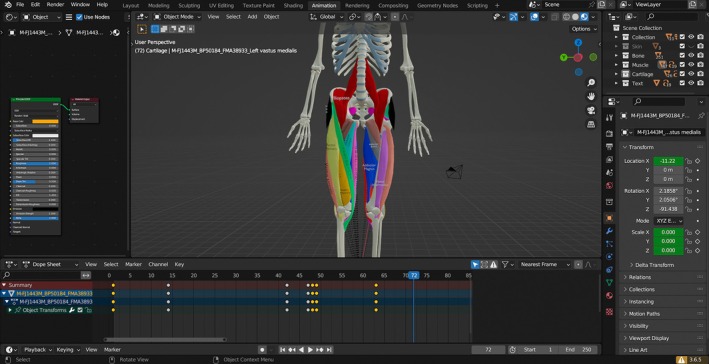
Blender's “animation” tab. Keyframes for the removal of superficial muscles are visible at the bottom of the screen. On the model, the superficial muscles have moved away from the model, and the deep muscles are now visible.

To achieve the goal of accurately representing musculoskeletal structures in 3D space, a method for viewing both the superficial and deep layers of muscles on the digital anatomical model was created (Figure 4). Using Blender's keyframe‐based animation, superficial muscles of the anterior left hip and knee (rectus femoris, vastus medialis, gracilis, adductor longus, and sartorius) were designed to move away from the model as one unit (accomplished by turning rectus femoris into the “parent” object, and having the other muscles follow its actions), at the user's input, before disappearing (accomplished by turning the X, Y, and Z dimensions for scale to zero). This allowed for greater visibility of the deep muscles (vastus intermedius, adductor magnus, and adductor brevis) while still placing them in the three‐dimensional context of the superficial ones.

**FIGURE 5:**
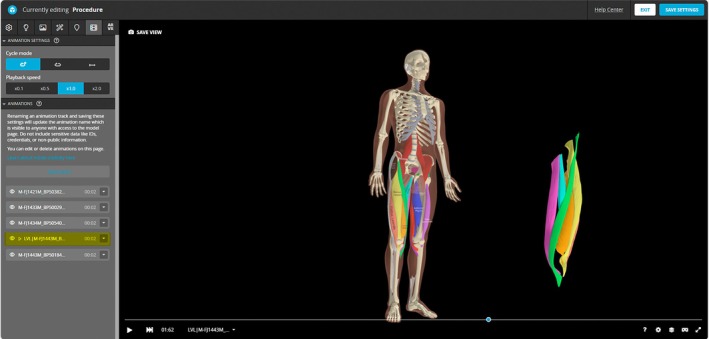
Selection of the correct animation pathway in Sketchfab. Background color has been adjusted to black.

The completed Blender model was exported as an FBX file, as this format allows for animation data to be uploaded to the Sketchfab platform. Uploading Blender exports to Sketchfab is completed via file selection. Within Sketchfab, the 3D settings of the model can be edited, allowing for a change in background and materials, and selection of the correct animation pathway. The background was set to black (HEX: 000000) and the opacity of the skin, which obstructed the rest of the model from view at 100%, was reduced to 10%. Subsequently, the correct animation pathway (highlighted in Figure 5) was selected, while the other options were deleted. The model was published with visibility set to “private” to prevent any external access to the model before the trial.

### Model evaluation: Context and participants

The participants were students at a large midwestern university, all of whom were enrolled in the Winter 2025 Musculoskeletal Anatomy class within the School of Kinesiology. The pilot trial was conducted over the first weeks of the semester, before the Musculoskeletal Anatomy course's first exam, while students were still learning fundamental anatomical terminology and the bony landmarks of the axial skeleton. The introduction of the muscular system occurs roughly halfway into the class; as such, the structures to be studied on the 3D model and textbook images were novel content to the students. The pilot trial was designed using the Canvas LMS to gather preliminary data on the extent to which students could improve their score from baseline on a mock exam comparable to the ones given in the Musculoskeletal Anatomy class; specifically, students completed a multiple choice, image‐based musculoskeletal anatomy examination after a brief (10 min) exposure to either textbook images or the 3D model. A brief design was chosen for this pilot trial to eliminate the confounding problem of students utilizing many different learning resources over the course of a semester and to avoid the ethical consideration of limiting their access to certain learning tools while in a graded classroom environment. Participation was optional for students in the Musculoskeletal Anatomy class, and a small amount of extra credit points (comparable to other “bonus” homework) was offered for enrolling—in other words, academic success in the course was not contingent on taking part in the trial.^22^ The pilot trial was deemed exempt from ongoing review by the university's Institutional Review Board (eResearch ID: HUM00262642). Additionally, students were informed that their participation in the trial was voluntary and that they were free to leave at any time without penalty.

### Pilot trial

**FIGURE 6:**
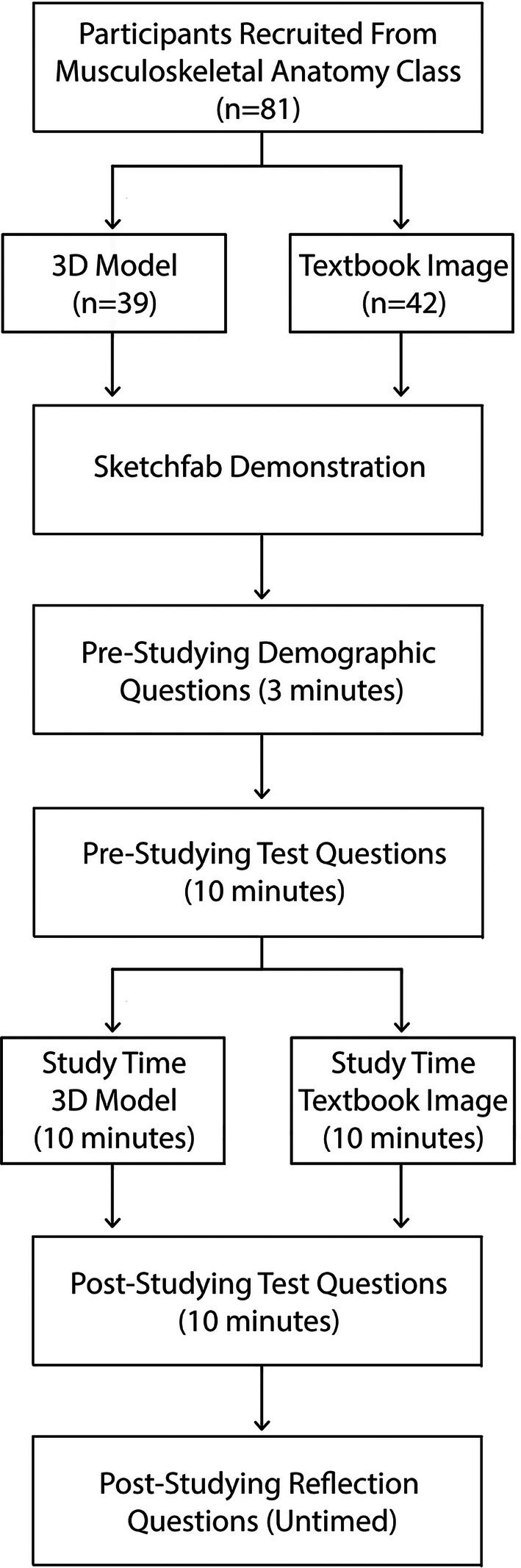
Flow chart of participants' progression through the pilot trial.

Students in the Musculoskeletal Anatomy class (*n* = 81) signed up to test the model in the pilot trial using an online spreadsheet with four available time slots (Figure 6). They were randomly assigned to either the 3D Model Group, “Group 1,” (*n* = 39) or the Textbook Image Group, “Group 2,” (*n* = 42) and subsequently joined the Group 1 or Group 2 Canvas page through a provided link or QR code. The trial was conducted at a computing lab on campus to ensure that all students in both groups completed the Canvas modules using the same technology, leveling the digital playing field. We expected that guaranteeing Group 1 students access to a mouse would make learning model navigation as easy as possible, which was especially important given the short duration of “Study Time.”

**FIGURE 7:**
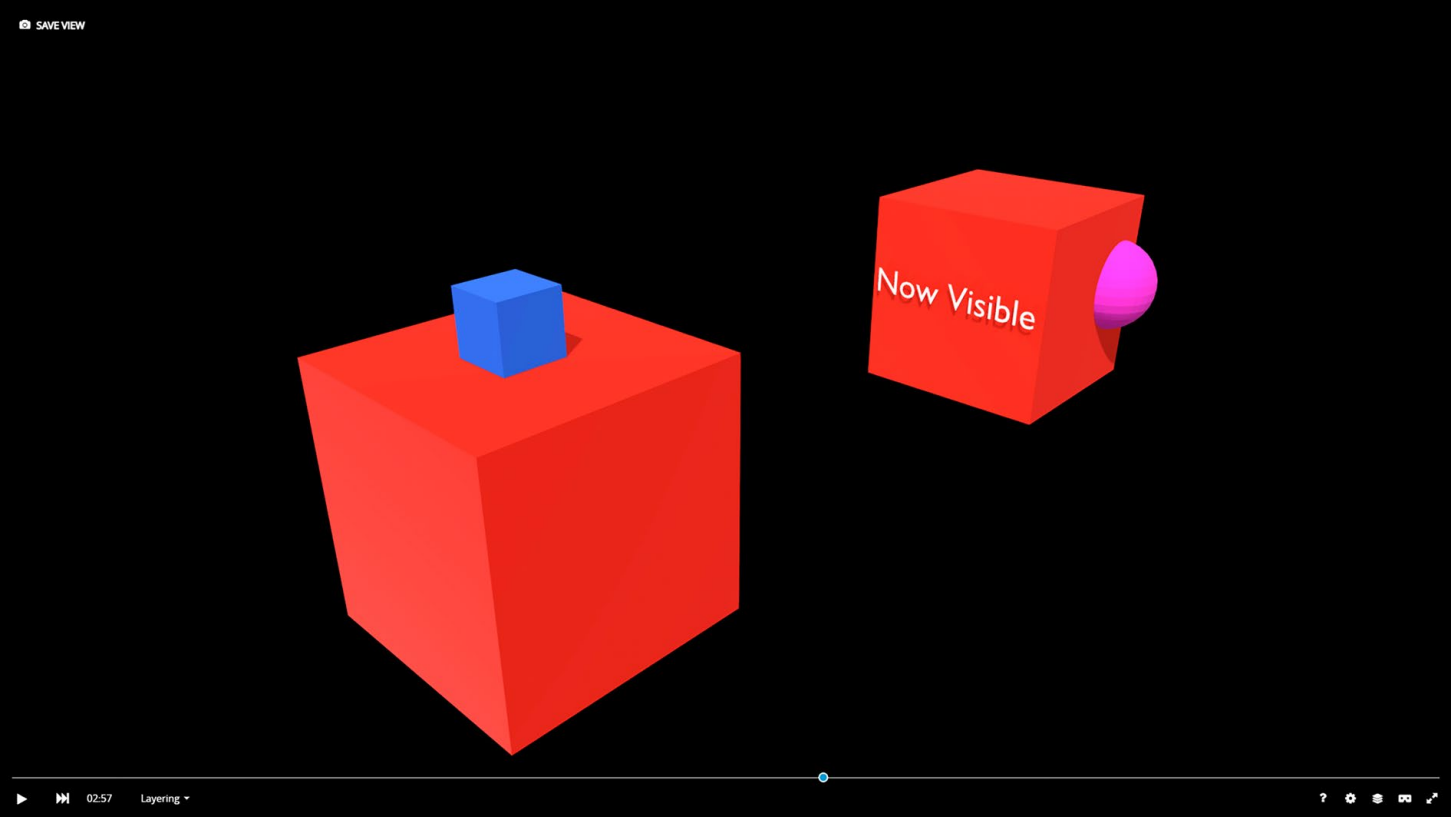
Sketchfab Demonstration model with hidden text “Now Visible” revealed.

The first module of both groups' Canvas course, “Sketchfab Demonstration,” contained a simple, animated 3D model (two cubes in an empty plane) on Sketchfab used for demonstration and practice (Figure 7). All students, regardless of group, followed a facilitator‐led demonstration about how to navigate Sketchfab's 3D space, learning how to control the progress of the demonstration's animation. Using the animation progress bar at the bottom of the screen, students manipulated the amount of space between the cubes—a skill which they would need to apply later in order to alternate between viewing the superficial and deep muscles of the model. All students had to enter the hidden message (“Now Visible”) into a text box within Canvas in order to proceed with the trial, ensuring that they had learned how to navigate Sketchfab. After the “Sketchfab Demonstration” module concluded, students could only receive assistance for technical issues and could not ask questions about the content they were studying.

Students then progressed to the “Pre‐Studying Demographic Questions” module, where they had 3 min to share their prior experience with anatomical education. Upon completion of the questionnaire, students were asked to wait until instructed to “flip” the digital page and move on to the “Pre‐Studying Test Questions” (synchronously taken by all present). Participants were given exactly 10 min to complete these twenty‐one multiple choice questions, which were the same for both groups, and each worth one point (for a maximum total score of twenty‐one points). Each question displayed an anatomical donor image of the hip and knee, along with arrows pointing to the tested muscle, which was highlighted for clarity. Anatomical donor images were used to avoid unfairly advantaging either group (e.g., by using a different textbook's images). After 10 min, the “Pre‐Studying Test Questions” Canvas module automatically closed, preventing students from taking further time on the questions. The facilitator then verbally instructed participants of both groups to progress to the “Study Time” module.

The “Study Time” module differed between the 3D Model Group and Textbook Image Group, containing either the 3D model or images from the course's textbook (*Kinetic Anatomy*), respectively.^23^ The students in the 3D Model Group had the model embedded on the “Study Time” page, allowing for easy access to the resource without having to leave the Canvas site. Students in the Textbook Image Group studied scanned textbook images from the computer screen. Students were given exactly 10 min to learn the muscles of the hip and knee using their assigned resource. After 10 min, the Canvas module automatically closed, preventing continued access to the resource.

Immediately following “Study Time,” students were instructed to proceed to the “Post‐Studying Test Questions.” Students had 10 min to complete the same twenty‐one questions on the pre‐test but presented in a different order. Participants then progressed to the “Post‐Studying Reflection Questions,” which gave them an opportunity to reflect on their experience with their assigned resource using a 7‐point Likert scale. There was also an optional free‐response section so that they could share how they felt about the experience. This section was not timed, and students were allowed to exit the trial once they confirmed that they had completed the module.

All data analyses were performed in Microsoft Excel 2025. An independent samples *t*‐test was used to compare the improvement in score by group between the Pre‐Studying and Post‐Studying Test Questions. An additional independent samples *t*‐test was used to compare the baseline scores of each group. The significance level was set at *p* < 0.05. Thematic analysis was conducted on free‐response feedback to compare resource perception between groups. These qualitative analyses were intended to contextualize the quantitative findings rather than contribute to theory.

### Demographic data

The Pre‐Studying Demographic Questions revealed strong baseline similarities between the 3D Model and Textbook Image Groups (Table 1). Both were mainly second year students (71.8%; *n* = 28 and 71.4%; *n* = 30, respectively) pursuing a degree in the School of Kinesiology (92.3%; *n* = 36 and 88%; *n* = 37). Additionally, the groups had comparable rates of prior academic experience in anatomy, physiology, or biology courses (82.1%; *n* = 32 of the 3D Model Group and 85.7%; *n* = 36 of the Textbook Image Group) and reported equal exposure to musculoskeletal anatomy through fitness and personal training (64.1%; *n* = 25 and 64.3%; *n* = 27). Finally, both groups reported low confidence in their knowledge of musculoskeletal structures of the hip and knee, with only 18% (*n* = 7) of the 3D Model Group and 21.4% (*n* = 9) of the Textbook Image Group agreeing to some extent with the statement, “I believe that I have a strong grasp of the musculoskeletal structures of the hip and knee.”

**TABLE 1:** Demographic differences between the 3D Model Group and Textbook Image Group.

Pre‐studying demographic characteristics				
Baseline characteristic	3D model group (*n* = 39)		Textbook image group (*n* = 42)	
	*n*	%	*n*	%
Class year				
Freshman	9	23	5	12
Sophomore	28	72	30	71
Junior	2	5	7	17
Senior	0	0	0	0
Degree/Program				
Applied exercise science	7	18	8	19
Biology	0	0	0	0
Biomedical engineering	1	3	2	5
Movement science	29	74	29	69
Undecided	1	3	2	5
Other	1	3	1	2
Previous anatomy classes				
Anatomy 303 (online)	1	3	0	0
Anatomy 403	2	5	3	7
Biology 225/226	0	0	2	5
Highschool A + P	20	51	19	45
Physiology 201	6	15	6	14
Other	12	31	17	41
No answer (none)	7	18	6	14
Other anatomy learning				
General interest/Self‐study	24	62	29	69
Physician shadowing	8	21	7	17
Internship/Research	5	13	8	19
Physical therapy shadowing	12	31	10	24
Online fitness	25	64	27	64
None of the above	1	3	1	2
Confident in hip/Knee anatomy				
Strongly agree	1	3	0	0
Agree	0	0	2	5
Slightly agree	6	15	7	17
Neutral	6	15	6	14
Slightly disagree	8	21	9	21
Disagree	14	36	14	33
Strongly disagree	4	10	4	10

### Test performance

A Jarque–Bera test was used to assess the normality of the data collected in this pilot trial. Neither the 3D Model Group (JB = 0.42, *p* = 0.81) nor the Textbook Image Group (JB = 2.4, *p* = 0.30) violated assumptions of normality and could be analyzed accordingly.

**FIGURE 8:**
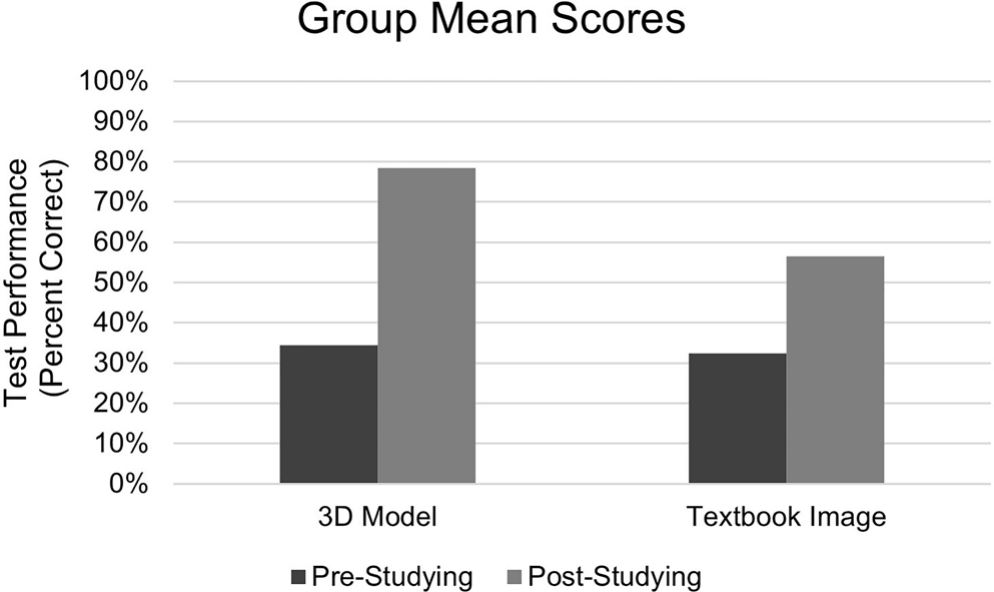
Scores on the Pre‐Studying and Post‐Studying Test questions compared by group.

There was a significant difference in the change from Pre‐ to Post‐Studying Test scores based on group (*t* = 4.924, *p* < 0.0001; 95% CI: 2.51, 5.91), such that the 3D Model Group (mean change = +9.26 points [+44.08%]) showed greater improvements on the test than the Textbook Image Group (mean change = +5.05 points [+24.04%]) (Figure 8). The mean score for the 3D Model Group on the Post‐Studying Test Questions was 16.46/21 points (78.39%), and the mean score for the Textbook Image Group was 11.86/21 points (56.46%). There was no significant difference (*p* = 0.5816) in score on the Pre‐Studying Test Questions between the 3D Model Group (mean = 7.21 points [34.31%]) and Textbook Image Group (mean = 6.81 points [32.43%]).

### Student perceptions

**FIGURE 9:**
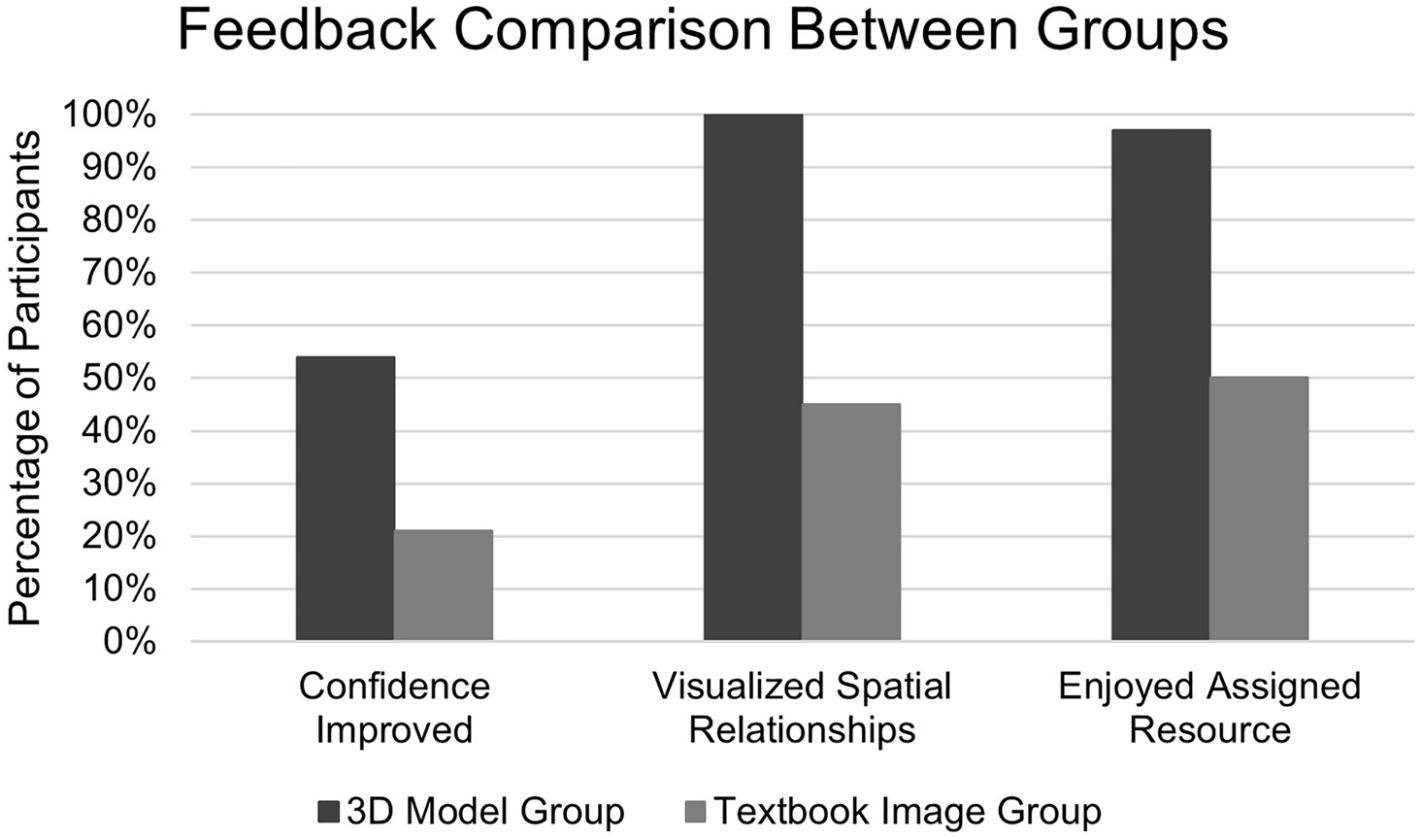
Comparison of the feedback from the 3D Model Group and Textbook Image Group. The 3D Model Group reported increased confidence from the Pre‐Studying to Post‐Studying questions, heightened ability to visualize spatial relationships between muscles, and greater enjoyment of their assigned resource than the Textbook Image Group.

Participants were asked to answer several questions about their experience with either the 3D model (*n* = 39) or textbook images (*n* = 42) (Figure 9). 54% (*n* = 21) of students in the 3D Model Group strongly agreed that their confidence in the assigned material had improved from the “Pre‐Studying Test Questions” to the “Post‐Studying Test Questions,” compared to 21% (*n* = 9) in the Textbook Image Group. 100% (*n* = 39) of the 3D Model Group agreed to some extent with the statement, “My resource helped me visualize the spatial relationship between muscles,” compared to 45% (*n* = 19) of the Textbook Image Group. Finally, 97% (*n* = 38) of students in the 3D Model Group agreed to some extent with the statement, “I enjoyed using my assigned resource,” compared to 50% (*n* = 21) in the Textbook Image Group.

The free‐response section within the “Post‐Studying Reflection Questions” allowed students to provide specific feedback about their assigned resource. An inductive thematic analysis was conducted to identify common themes. Coding students' comments revealed qualitative differences between groups that reinforced the quantitative findings. The themes (resource sentiment, desire for integration, translatability, and contextual clarity) also provided further insight into which aspects of their assigned resource students felt most strongly about. Two members of the research team independently categorized each participant's comments, resolving any initial discrepancies through refining the criteria for coding.

Students in the 3D Model Group were more likely to express a positive sentiment about their resource with representative statements such as “It helped me memorize and understand the muscles much better than a textbook.” In particular, they noted that the presentation of information was clear and facilitated their learning following the baseline assessment. In comparison, students in the Textbook Image Group were more likely to express mixed sentiments with representative quotes such as “Photos were cleanly labeled, but it was difficult to see the layers of muscle” and “I could hardly identify any muscle that I remembered from the study material.”

Several members of the 3D Model Group mentioned a desire to use their resource again, with statements such as “I hope we can integrate the 3D model in the course” and “I enjoyed using this tool and hope I can use it in the future to study.” In contrast, members of the Textbook Image Group noted that they would have preferred to try the 3D model instead of using the images they were provided, with feedback such as “3D models would be a more interactive and engaging experience even though I know how to use these diagrams.”

Students in both groups acknowledged having some amount of difficulty translating their resource to the donor image‐based Post‐Studying Test Questions. One student in the 3D Model Group stated that “In the quiz post‐studying, it was easier to answer the questions but it was still hard because it looked somewhat different than the study mode.” Students in the Textbook Image Group stated that the textbook images “looked so different from an actual human body” and “would have been more effective if they were more similar to the view of the test questions.” However, while both groups reported difficulty with translatability, 33% (*n* = 14) of students in the Textbook Image Group expressed this sentiment, compared to only 15% (*n* = 6) in the 3D Model Group.

Finally, several students in the Textbook Image Group reported challenges with resource clarity and context, stating that “it was difficult to tell the difference between muscles in these pictures” and “using this source did not allow for a spatial arrangement between structures to be shown.” In contrast, students in the 3D Model Group highlighted the clarity of spatial relationships as a benefit of their resource, with representative feedback such as “The model made it easier to visualize which muscles were superficial vs deep than a 2D image would have” and **“**I liked being able to manipulate and separate all of the different layers.” Overall, 3D Model Group members frequently emphasized the clarity produced by the model's distinct layers and spatial manipulability.

## DISCUSSION

A highly accessible, 3D, digital musculoskeletal anatomy model was successfully developed and uploaded to the Sketchfab platform according to the protocol described above. The preliminary data collected in our pilot trial suggest that studying musculoskeletal structures from the 3D model was more effective than using textbook images in cases of brief exposure when comparing image‐based multiple choice exam performance. Additionally, the qualitative measures revealed a preference for using the 3D models over textbook images, with Group 1 reporting higher confidence in the material, understanding of muscles' spatial relationships, and overall enjoyment of their resource.

While individual designs report different outcomes on the effectiveness of 3D digital models, a 2015 meta‐analysis by Yammine and Violato found that, across thirty‐six studies, students who used three‐dimensional visualization technology (3DVT) exhibited, on aggregate, superior factual anatomical knowledge, spatial knowledge, and resource satisfaction compared to those who used 2D materials, such as textbooks.^17^ These findings are in line with the data presented here and form a compelling argument for the implementation of 3DVT, such as 3D digital models, into anatomy curricula. Additionally, the studies included in the meta‐analysis varied in length, with benefits to using these resources found in both short (hours) and longer term (week‐long or semester length) designs.^24^, ^25^ However, there are nuances within the literature that should be explored to understand how 3D digital models can be used most effectively.

One study on 3D digital models of vascular anatomy used throughout the semester in medical school curricula found that second semester students with access to the models performed significantly better than their matched peers from the previous year.^14^ However, fifth semester medical students, further into their studies, performed equally to their matched peers who didn't have access to the resource. The models' positive impact on performance early in students' medical education was not maintained in upperclassmen, suggesting that those still building their foundational anatomical knowledge and study skills may derive greater benefit from these resources. In other words, digital learning resources such as the one we have developed here may be most beneficial for those early in their anatomical education and can serve as a novel supplement to encourage student engagement.

Another study on 3D digital models found that medical students completed either a hand or foot anatomy exam more quickly and with higher scores when they studied from models instead of atlas images.^26^ The authors proposed that their models' “search” feature (allowing students to quickly find structures by name) contributed to this improvement in both speed and knowledge acquisition, while also highlighting the decrease in cognitive load that comes from removing the mental translation of 2D images into 3D space. Interestingly, junior and advanced medical students had a similar increase in score, but the article acknowledges that considerable time had passed since the advanced students studied hand and foot anatomy, which is reflected in their baseline scores being equal to the first‐time learners; therefore, we suggest that these results do not necessarily conflict with the idea that novice learners stand to gain the most from digital learning resources.

While much of the research on 3D digital learning resources is compelling, it is worth exploring instances where these tools failed to be more effective than traditional educational methods. One brief (<1 h) study of similar design to our pilot trial compared the effectiveness of a 3D‐printed equine foot model to a digital model and textbook images, and found that third‐year veterinarian students studying from the printed model outperformed those in both the digital and textbook image groups, who performed equally.^12^ This finding aligns with expectations that a tangible experience, such as that provided by anatomical donor dissection, remains the gold standard in anatomical education. However, students in the 3D‐printed model and digital model groups reported significantly greater enjoyment of their resource and increased confidence in their anatomical understanding of the equine foot compared to the textbook image group. In a similar vein, both Keedy et al. and Tan et al. found no significant difference on post‐studying exam scores between their respective 3D digital model and traditional resource (2D images) groups.^27^, ^28^ However, despite the lack of quantitative improvement over a short‐term studying window, students still reported greater enjoyment of the digital resource in both studies, while maintaining comparable scores. Performance aside, developing resources that students enjoy using is a highly desirable outcome outside the context of a structured and time‐bound research trial, possibly leading to greater engagement and time spent independently studying class material over the course of a semester.

With the historical literature in mind, along with the data presented here, there is evidence to suggest that 3D digital models can be an effective resource for anatomical education. Further research is needed to elucidate the precise mechanism behind this connection; although, as noted above, some studies have shown that digital learning tools may decrease cognitive load and working memory demands, despite the learning curve involved in navigating the resource.^26^, ^29^, ^30^ Another sensible explanation may lie in the fact that 3D digital models translate better to real‐world anatomy, which is neither two‐dimensional nor static. While both the 3D model and textbook images bear limitations in applicability to donor images, the ability to manipulate a 3D model, viewing the complex layering of the body's systems from different perspectives and within the context of relatively superficial or deep structures is undoubtedly beneficial for learning. Additionally, students' increased enjoyment of digital learning tools relative to textbook images may play a role in their effectiveness, prompting strong engagement and personal initiative to explore the novel resource. In sum, using both the historical literature and the findings presented here, we suggest that 3D digital models can be most effectively implemented into anatomical education when they (1) are used early in a student's anatomical education, (2) have convenient and innovative features such as distinct colors, animation, or a “search” function, (3) are perceived as highly enjoyable to use, and (4) are used in conjunction with other effective learning modalities, such as donor dissection.

The circumstances of the 3D model's development are important to highlight. No members of the research team earned a degree in a technology‐intensive field. Assembling the model was a joint effort consisting of reading online resources, watching video tutorials, and generally experimenting in Blender. The result was the creation of a model with no associated cost aside from the time investment to find the open‐source files on BodyParts3D and to learn the basics of Blender. Sketchfab, while having paid options, is free to use as well, in addition to being browser‐based and usable on any type of computer, phone, or tablet, even without an account. The research team created a resource that is accessible to any student with a device that can connect to the internet. While more comprehensive models with unique features exist (such as Elsevier's Complete Anatomy, which contains information about origins, insertions, and muscle actions), there are few whose creation and use are entirely free to both the developers and students– and that are curated to a specific curriculum. We hope that the data presented here demonstrate the benefits of using open‐source tools to ensure that all students have access to resources that enhance their learning.

Our design is not without limitations. Ideally, participants would be assigned to only one of the resources over the course of an entire semester to minimize the effects of any initial digital learning curve and to compare factors such as long‐term retention of information between groups. However, this would be unfair to the students who wish to succeed in the class by using all resources available to them. We therefore hope that the preliminary findings presented in this article warrant the undertaking of further research with more rigorous designs which take place over longer periods of time, potentially in a non‐academic environment, so as to limit these ethical concerns. Second, while the 3D model contained many different features, including color, annotation, and animation, we were unable to conclusively identify which specific features of the model were most advantageous to students although this is an interesting direction for future study. Additionally, while participants were instructed to give their best effort on both the Pre‐ and Post‐Studying Test Questions, they were also informed that they would receive the extra credit offered for the assignment regardless of their score. This led to some students—particularly those in the Textbook Image Group—not using the full 10 min allotted for the test questions, making room for careless mistakes or lack of full effort. Finally, our design only explored the difference between 3D digital models and textbook images used independently of each other. We believe that the most effective way to implement digital resources such as the one developed here into anatomy curricula is in conjunction with other well‐established learning modalities, offering students a variety of content to engage with.^5^ Future studies should be conducted to assess whether the use of 3D digital models in conjunction with other resources, including textbook images, facilitates greater exam performance than using only one of the resources.

## CONCLUSION

This article described the creation of a three‐dimensional musculoskeletal anatomy model, showing the potential for creating digital learning resources with no associated costs for the creators or students. The findings of our subsequent pilot trial supported the use of 3D models for first‐time learners of musculoskeletal anatomy. While the design did have limitations in its brevity and lack of a dual‐resource group, the data still indicate the viability of the model. Future studies with more rigorous designs should further investigate the effectiveness of 3D models—especially those made with open‐source materials—in anatomical education and should aim to elucidate the specific mechanisms behind their effectiveness.

## AUTHOR CONTRIBUTIONS

**Alexander H. Safir:** Conceptualization; investigation; writing – original draft; writing – review and editing; visualization; methodology; formal analysis; data curation; supervision. **Michele M. Bird:** Supervision; resources; investigation. **Mary E. Orczykowski:** Project administration; methodology; writing – review and editing; supervision; conceptualization; investigation.

## FUNDING INFORMATION

This study was undertaken at no cost to either the authors or the University of Michigan.

## CONFLICT OF INTEREST STATEMENT

None of the authors has a conflict of interest to disclose.

## ETHICS STATEMENT

This study was determined to be exempt upon review by the University of Michigan's Institutional Review Board (HUM00262642). Participation in the study was voluntary, and participants were informed that they were free to leave at any time without penalty.

## Data Availability

Anonymized raw data for the experimental groups' scores and responses are available upon request.
